# Challenges in implementing new nursing qualifications (Regulation 174) in South African public nursing colleges: Principal perspectives

**DOI:** 10.4102/curationis.v48i1.2544

**Published:** 2025-06-26

**Authors:** Magdeline N. Poto-Rapudi, Thembekile E. Masango, Masenyani O. Mbombi

**Affiliations:** 1 Department of Health Studies, College of Human Sciences, University of South Africa, Pretoria, South Africa; 2 Department of Nursing Sciences, Faculty of Health Sciences, University of Limpopo, Polokwane, South Africa

**Keywords:** challenges, public nursing colleges, implementation, new nursing qualifications, higher education institutions, accreditation, nursing education institution

## Abstract

**Background:**

The criticism levelled against the legacy nursing programmes has transformed the health education and training system and the preparation of nursing students to meet society’s needs. The prevailing practice in nursing education (NE) change is orientated towards increasing professionalisation, which necessitates expanding nursing programmes to provide universal health coverage. The South African Nursing Council (SANC) has mandated NE institutions to curriculate and institute the new qualifications in nursing.

**Objectives:**

This article explores the challenges attendant to the implementation of the new qualifications in nursing at selected public nursing colleges (PNCs) in North-West, Gauteng, Limpopo and Free State provinces.

**Method:**

A qualitative research design approach was adopted, with semi-structured interviews conducted with 13 purposively sampled participants that were transcribed verbatim. Tesch’s eight-step data analysis method was utilised for the development of the study findings framework.

**Results:**

The findings revealed challenges such as lack of essential human and infrastructural resources, infrastructural and material limitations, logistical and service constraints and mixed perceptions on the support system available to PNCs ahead of implementing the new qualifications in nursing.

**Conclusion:**

The perspectives of the principals’ show various challenges that can be categorised into human, institutional, and structural factors. These challenges suggest a need to strengthen the academic support and collaboration between internal and external stakeholders of NE institutions for effectively implementing the new programmes in nursing.

**Contribution:**

The study findings create awareness for the SANC, Council on Higher Education and Department of Health about the progress of the new nursing programme implementation.

## Introduction

There is a pressing need to transform South African nursing education (NE) and training. Key stakeholders in this transformation include the principals of nursing education institutions (NEIs), the South African Nursing Council (SANC) and the Council on Higher Education (CHE) (Direko & Davhana-Maselesele 2017). The current study examines the challenges faced by NEI principals in implementing new nursing qualifications mandated by the SANC. This transformation is crucial for preparing nursing professionals to address global health demands, alleviating the ongoing nurse shortage, and curbing migration of nurses to countries that offer better salaries (Armstrong & Rispel 2015; Bezuidenhout, Human & Lekhuleni 2013; Blaauw, Ditlopo & Rispel 2014; Crowley & Daniels 2023; Matlakala 2016). It is also essential to reduce the high rates of diseases such as human immunodeficiency virus (HIV) and acquired immune deficiency syndrome (AIDS), and improve maternal and infant mortality rates (Joseph & Bomela 2022; Statistics South Africa 2015). Given the nursing profession’s growth opportunities, competitive salaries and career stability (Flaubert et al. 2021), the continuous transformation of NE is necessary to address these challenges and the demand for skilled nurses.

According to Tamata and Mohammadnezhad (2023), addressing the shortage of qualified nursing forces across many regions and countries contributes to producing more skilled nursing professionals to meet the growing demand for healthcare services. Politically, the healthcare industry is a significant contributor to the economy, and expanding NE subsidises the required national economic growth by creating job opportunities and supporting the healthcare sector (Boyce & Brown 2019). Also, political leaders in South Africa direct the nursing profession’s management (Blaauw et al. 2014; Zwane & Mtshali 2019). Therefore, transformation is done for the compliance with the national political structure.

Crowley and Daniels (2023) uphold that reform of the NE programmes in South Africa is significant for the production of quality nursing professionals and that quality health services are sustainably delivered in the country. Accordingly, the transformation of nursing programmes contributes to improving healthcare quality by producing knowledgeable graduates who are well prepared and equipped with the skills necessary for providing safe and effective care to patients (Fawaza, Hamdan-Mansourb & Tassi 2018). Notably, transforming NE requires adequately resourced NEIs in order to accomplish their mission of educating responsive, accountable, ethical and knowledgeable nurses (Coetzee 2019). The success of transforming NE is premised on well-defined standards for national professional education, unambiguous accreditation guidelines, increased capacity for nurse educators and quality maintenance (Gorski et al. 2015).

The successful transformation of programmes for NE and training is enhanced by effective and transparent curriculum development within the NEIs, the accreditation process by the SANC and the CHE and effective implementation of the programme by principals and nurse educators (Maree, Yazbek & Leech 2018). Regrettably, the transition to new nursing qualifications within the aforementioned three stakeholders from 7 years ago has temporarily inhibited nurse training because of implementation-related challenges affecting the new programmes in nursing, which frustrated principals and nurse educators in NEIs.

Notwithstanding the blueprint provided, South Africa continues to encounter challenges linked to transforming NE (Blaauw et al. 2014). These challenges are fundamentally based on overdue accreditation of the governance and leadership structures in the country’s NEIs. Some of these challenges from a narrative and literature review perspective include tedious waiting periods for programme reform and accreditation by internal and external stakeholders (Maree, Yezbek and Leech (2018), the stringent requirements by CHE, unsynchronised processes between internal and external stakeholders, delayed guidance on sequencing the accreditation process and the different approval requirements for accreditation by external stakeholders with inconsistent communication and feedback to NEIs (Crowley & Daniels 2023). The perspective of one of the listed stakeholders – the principals from the NEIs – remains understudied within the selected provinces of South Africa.

## Background

*The Constitution of the Republic of South Africa (Act No. 108 of 1996)* enjoins the Minister of Education to be responsible for overseeing a single and coordinated system of higher education (South Africa 1996). The Minister of Higher Education is also charged with accrediting any NEI to become a higher education institution (HEI) and registration of private nursing HEIs with the Department of Education (DoE) (South Africa 1996, 2008). As such, the nurses’ education and training must take place in a policy environment defined by legislation for both higher education and health as well as by the shared responsibility and monitoring of the Department of Higher Education and Training (DHET) and the National Department of Health (NDoH) (Republic of South Africa 2019). For example, the *Higher Education Act (No. 101 of 1997 as amended)* governs the academic programmes of all HEIs, including NEIs (South Africa 1997), by regulating the functions of the CHE and the SANC.

The CHE plays a significant role in developing and managing South Africa’s Higher Education Sub-Framework (HEQSF). The HEQSF provides a structured framework for the organisation, quality assurance and classification of higher education qualifications. Therefore, the HEQSF stipulates the requirements of the CHE’s required programmes for registration, accreditation and approval by the South African Qualification Authority (SAQA) (South Africa 2014). All NEI academic programmes ought to align with the HEQSF’s requirements.

It is expected of NEIs to provide the SANC and CHE with their evaluated curriculum at the same time (Zwane & Mtshali 2019). The SANC is authorised by the SAQA to serve as an education and training quality assurance (ETQA) organisation for nursing qualifications as stipulated in *Section 5 of the SAQA Act (No. 58 of 1995)*.

Thus the SANC regulates, endorses and directs new nursing curricula and programmes or qualifications (SANC 2013b). Moreover, the SANC prescribes and manages standards for NE and evaluates the education and training for nurses in respect of the needs of the country. To fulfil the mission of NE transformation, the SANC has to consider the provisions of the law regarding certification, accreditation and sustenance of applicable national standards for education and training and also support nursing with regard to policy execution for NE and training. Therefore, SANC and CHE support is essential for programme curriculation and accreditation to NEIs. Notably, the essential CHE and SANC accreditation support by cluster evaluations and training evaluators and cluster yielded little progress regarding the implementation of nursing programmes.

In South Africa, the SANC and the NDoH govern education, training and practices in nursing. The NDoH formulates and implements health policies and programmes at the national level, including policies for NE, training and practice in order to address the population’s healthcare needs effectively. The NDoH is tasked with alignment of nursing practices in education and training with identified priorities in healthcare delivery, ensuring that the acquired qualifications are in synchrony with the scope of practice and promulgating the necessary regulations for NE and training regulations (Makhanya 2018). In 2008, the NDoH published strategies for nursing in order to guide the provision of sufficient training proportional to the country’s needs (Bruce, Klopper & Mellish 2011). In 2013, the NDoH further published regulations for transforming legacy programmes in public nursing colleges (PNCs) for preparation for new programmes to be submitted to SANC and CHE by 2015. The PNCs operate under the fiat of the NDoH, which provides support for funding student posts and other related resources such as equipment, human resources and buildings for implementing academic programmes effectively.

Furthermore, PNCs are urged to identify programmes that address national interests and needs and also regulate minimum education and training stipulations for the general nurse category (R171 of 8 March 2013) (SANC 2013a). Notably, the envisaged R174 programme is not prioritised by many PNCs as they yield more graduates than universities. Accordingly, PNCs have started offering the R171 education and training programme for students aspiring to register as general nurses, as the preferred level for professionalising nursing (SANC 2013a). A new 4-year bachelor’s programme was identified and approved for minimum requirements in professional midwifery (R174) (SANC 2013b). The changes in categories required all NEIs’ alignment of nursing qualifications.

### Problem statement

The Minister of Education oversees the implementation of a unitary higher education system for increased accessibility. As such, the SANC and CHE require the public nursing education institutions (PNEIs) to be well resourced for the R174 programme and its aim of developing professional nurses. However, the progress of PNEIs in developing and implementing the new qualifications remains uncertain, posing a risk to health workforce performance and nurses’ professional status.

### Purpose of the study

The purpose of this study is to explore the challenges attendant to the implementation of new nursing qualifications in selected PNCs in North-West, Gauteng, Limpopo and Free State provinces of South Africa.

### Contribution to the field

The results of the study offer a current assessment of how well the new nursing programmes are being implemented at the NEIs. When creating support measures to guarantee the successful implementation of new nursing programmes, the difficulties faced by NEI principals could serve as a framework for assessing their implementation. This study will add to the body of knowledge on NE reform in South Africa, based on its focus on the PNEIs’ readiness to move to higher education in the quest to deliver National Qualifications Framework (NQF)-compliant credentials.

Therefore, the results of the study may also reveal the extent of the South African PNEIs’ preparedness to offer new R174 qualifications. These suggestions apply to South African public nursing schools, as well as other nations that are reforming NE. Nursing colleges struggle with ageing nurse educators, causing a loss of competence and institutional memory. Programmes are aimed at developing capacity, but implementation is sparse. Collaboration and professional development are needed.

## Research methods and design

### Study design

A qualitative exploratory-descriptive research design was adopted in this study, which enhanced the authors’ in-depth exploration of the phenomenon of interest (Marshall & Rossman 2016); that is, the challenges that principals of selected NEI’s experience when implementing the new qualifications in nursing.

### Setting

We selected a total of 13 public NEIs in North-West, Free State, Gauteng and Limpopo provinces. These four provinces were conveniently selected by the primary author based on their accreditation status to offer R174 (Makhanya, Matahela & Buthelezi 2022). As depicted in Table 1, Gauteng and Limpopo provinces have the majority of four principals, followed by three principals in Free State province and North-West province with only two principals.

**TABLE 1:** Demographic characteristics of the selected public nursing colleges.

Provinces	PNC’s names	College capacities	Principals
North-West	PNC 1	600–699	1
North-West	PNC 2	700–799	1
Free State	PNC 3	900–999	1
Free State	PNC 4	700–799	1
Free State	PNC 5	600–699	1
Limpopo	PNC 6	900–999	1
Limpopo	PNC 7	900–999	1
Limpopo	PNC 8	500–599	1
Limpopo	PNC 9	900–999	1
Gauteng	PNC 10	1200–1299	1
Gauteng	PNC 11	1200–1299	1
Gauteng	PNC 13	300–399	1
Gauteng	PNC 12	1300–1399	1

**Total**	**-**	**-**	**13**

Source: Poto-Rapudi, M. & Masango, T.E., 2021, ‘Development of a Support Model for the Implementation of the New Nursing Qualifications in South Africa’, Doctoral thesis, University of South Africa

PNC, public nursing colleges.

The multiplicity of study sites provided room for the triangulation of data and assurance of the reliability and validity of the findings (Noble & Heale 2019) from principals across rural and urban NEIs. The gathering of data from widely spread sites also enhanced the quality of the findings, which significantly reduced errors and possible distortion (Noble & Heale 2019). All colleges across the provinces are involved in the implementation of the new qualifications.

### Study population and sampling strategy

The study population are the nursing students, their lecturers and principals at public and private nursing institutions in South Africa. However, the targeted population are only the 13 principals of the chosen PNEIs where the new R174 qualifications would be implemented. The non-probability purposive sampling strategy was applied to sample the 13 principals based on their roles within the PNEIs and their knowledge and experiences in designing and implementing nursing programmes. The chosen sampling method enabled the primary author to provide equal or guaranteed chances for the principals’ selection and involvement in the interviews (Grove, Gray & Burns 2015; Moule & Goodman 2014).

### Data collection

The principals from the selected sites were recruited with the help of the respective colleges’ research coordinators. The study’s objectives, potential benefits and voluntary nature of involvement were all explained by the study’s principal author. Data were collected from 13 principals of the selected PNEIs using a semi-structured interview with a guide. The primary author, as an experienced researcher in conducting interviews, took the lead in conducting semi-structured interviews, which lasted between 35 and 60 min. Some of the questions posed to the principals include ‘Please describe challenges you experience as a college to implement R174 programme’. Also, the principals were asked to describe the type of support provided to them by the accrediting stakeholders; that is, the SANC, CHE and provincial DoH.

The semi-structured interviews preceded the open-ended questions concerning the PNEIs’ human resources, clinical placement, student profile, library facilities, programme design and infrastructure. These questions enabled participants’ spontaneous and verbatim expression, which advanced the gathering of rich information in conjunction with the probing questions. In addition to the interviews, the primary author utilised observational notes for capturing the critical aspects emanating from the participants’ non-verbal cues and communication for further clarification. A digital voice recorder was utilised to record all of the interview sessions in order to obtain and preserve data in its original and uncontaminated state or form (Moule & Goodman 2014). Data saturation was reached when the primary author’s interview-based qualitative data reached closure or redundancy as no additional information could be acquired (Polit & Beck 2017). Accordingly, data saturation served as the foundation for sample size.

### Data analysis

The primary author singularly analysed the transcribed audio-recorded verbatim statements of the sampled principals in Microsoft Word. Thereafter, the researcher undertook the following analytic approach as proposed by Tesch (Creswell 2014; Grove, Burns & Gray 2013):

- Meticulous reading of all transcripts in order to obtain a clearer picture;
- Carefully noting important thoughts and underlying meanings in every transcript;
- Listing all significant topics and statements in the transcripts and grouping those that are similar, novel and obsolete;
- Abbreviating and coding the significant topics and statements in relation to segments of relevant texts;
- Describing and translating the main topics into categories;
- Reducing the categories in terms of topically related groups;
- Alphabetically listing and abbreviating each category
- Assembling data categories and performing initial analysis.

### Ethical considerations

Ethical clearance to conduct this study was obtained from the University of South Africa Research Ethics Committee (reference no: HSHDC/746/2017). The primary author undertook the study only after written ethical approval was formally granted by the Health and Research Ethics Committee (HREC) in the Department of Health Studies at the University of South Africa (UNISA). Each of the selected provinces’ Department of Health and PNC principals also approved the study to be conducted at their colleges and/or PNEIs. The principals’ right to self-determination was maintained, including uncoerced participation and withdrawal from the interviews whenever they perceived a transgression of their rights. The authors selected and treated the principals of PNEIs fairly and ensured anonymity by requesting the principals not to provide names or identifying information during the study. The authors further informed the principals about the study’s significance and purpose, after which they then signed informed consent prior to their participation. The interviews themselves took place in a private, pre-booked room that was not exposed to external disturbances.

There were no anticipated risks arising from the principals’ involvement in the study. The benefit of the principals’ involvement is that they would be able to provide updates on the progress of the new R174 programme’s implementation at South African PNEIs.

## Results

### Demographic profile

Table 2 captures the demographic characteristics of the participants in respect of their gender, nationality, race, age, educational qualifications, employment status and years of management experience.

**TABLE 2:** Demographic characteristics of the participants.

Criteria	Characteristics	Frequencies	Percentage
Gender	Female	11	84.61
	Male	2	15.38
Nationalities	South African	13	100.00
	Non-South African	0	0.00
Races	White people	1	7.69
	Black people	11	84.60
	Coloured people	0	0.00
	Indian people	1	7.69
Age categories (years)	35–40	0	0.00
	41–45	1	7.69
	46–50	3	23.00
	51–55	2	15.38
	56–60	4	30.76
	61–65	3	23.00
Qualification types	Postgraduate diploma	10	76.92
	Master’s	2	15.38
	PhD	1	7.69
Employment status	Permanent	12	92.30
	Contract	1	7.69
Years of experience in managing the institution	0–5	0	0.00
	6–10	2	15.38
	10–15	5	38.46
	16–20	6	46.15

Source: Poto-Rapudi, M. & Masango, T.E., 2021, ‘Development of a Support Model for the Implementation of the New Nursing Qualifications in South Africa’, Doctoral thesis, University of South Africa

PhD, Doctor of Philosophy.

Extrapolated from Table 1 is that 84.61% (*n* = 11) of the participants are female, and 15.38% (*n* = 2) are male. Additionally, all participants (100%, *n* = 13) are South African, most of whom (84.61%, *n* = 11) are Black, while 7.69% (*n* = 1) are White and 7.69% (*n* = 1) are Indian. Concerning the age range, most (30.76%, *n* = 4) were aged 55–60 years, while 23% (*n* = 3) were 46–50 years old and 23% (*n* = 3) were aged 61–65 years. Moreover, 15.38% (*n* = 2) of the participants were aged 51–55 years, while 7.69% (*n* = 1) were between 41 and 45 years in age.

Most of the participants (76.92%, *n* = 10) possessed postgraduate qualifications, while 15.38% (*n* = 2) possessed master’s qualifications, and only 7.69% (*n* = 1) possessed doctoral qualifications. Concerning their employment status, most participants (92.30%, *n* = 30) were on permanent employment, while 7.69% (*n* = 1) participants were on temporary or contract employment. Furthermore, most of the participants (46.15%, *n* = 6) had acquired management experience of 16–20 years, while 38.46% (*n* = 5) possessed 10–15 years’ management experience and 15.38% (*n* = 2) possessed management experience of 6–10 years.

### Themes and sub-themes

The primary author used an independent coder to confirm the veracity and relevance of the emerging themes and sub-themes concerning the challenges experienced by principals in implementing the new qualification programmes. In that regard, the participants mentioned that the challenges that they experienced related to human resources, logistics and services, as well as infrastructural and material constraints relating to the new qualifications programmes and their implementation. Table 3 depicts an overview of the themes and related sub-themes:

**TABLE 3:** Overview of themes and related sub-themes.

Themes	Sub-themes
1. Human resource challenges	1.1 Shortages of support staff and qualified lecturers 1.2 Disjuncture of old and new posts because of deficient funding and existing moratorium 1.3 Inadequate OSD and its effects on recruitment of nurse educators 1.4 Lack of clinical mentors/educators
2. Infrastructural and material constraints	2.1 Infrastructure-related problems 2.2 Material-related problems 2.3 Technology-related problems to realise 4IR
3. Clinical services and logistical problems	3.1 Challenges induced by MIS in relation to implementing the new qualifications 3.2 Institutional challenges at initiation and working stages of curriculation of new programmes
4. Mixed perceptions concerning the PNC support system for preparing the new qualification implementation	4.1 Poor support from accreditation bodies

Source: Poto-Rapudi, M. & Masango, T.E., 2021, ‘Development of a Support Model for the Implementation of the New Nursing Qualifications in South Africa’, Doctoral thesis, University of South Africa

OSD, occupation-specific dispensation; PNC, public nursing college; MIS, management of information systems; 4IR, Fourth Industrial Revolution.

### Theme 1: Human resource challenges in the implementation of new qualifications

Participants identified human resource challenges in NE, including a shortage of qualified lecturers, limited student mentors and support staff for new qualifications, as well as a lack of occupational-specific dispensation (OSD), which may hinder interest in this field.

#### Sub-theme 1.1: Lack of or limited number of qualified lecturers and support staff

Furthermore, there was an indication of a shortage of support staff and qualified lecturers to facilitate the implementation of the new qualifications, as corroborated in the following participant statements:

‘The CHE identified significant challenges due to the lack of clinical and educators with a Master’s degree.’ (Participant 8, 61 years, female)

‘We have challenges in preparing for the Bachelor’s Degree because the offering of this programme will require the educators to qualify for that …’ (Participant 10, 64 years, female)

#### Sub-theme 1.2: Creation and application of old and new posts problematic resulting from the existing moratorium and lack of funds

The participants alluded to the disjuncture of old and new posts because of deficient funding and existing moratoriums, both of which had a bearing on the new qualifications’ implementation. The participants expressed this view, thus:

‘The recruitment moratorium has been lifted, with 11 posts advertised for two colleges, and the Provincial Health Department is awaiting approval.’ (Participant 1, 46 years, female)

‘We do have nine vacant posts … Most personnel are lost due to retirement age, and others adventuring into new challenges …the HR processes delay the appointment.’ (Participant 4, 53 years, female)

‘Retiring staff not being replaced, and a lack of readiness for other programmes …’ (Participant 7, 48 years, female)

#### Sub-theme 1.3: Lack of occupation-specific dispensation affecting recruitment of nurse educators

The participants indicated staff-related problems that could affect the implementation of new qualifications, such as lack of OSD at PNCs, leading to poor interest by nurse educators. These views were expressed by the participants as follows:

‘The college faces challenges in recruiting and retaining educators due to the OSD salary structure, which may lead to job losses and potential career growth in clinical department promotions.’ (Participant 8, 61 years, female)

‘The main challenge is the salary structure, as experienced staff are not recruited due to the higher salary offered by the OSD.’ (Participant 13, 58 years, male)

‘However, our candidates do not accept the offer because of the salary structure … It is frustrating as we cannot change the OSD policy. This affects the staff as everybody is stretched.’ (Participant 11, 46 years, female)

#### Sub-theme 1.4: Lack of clinical mentors or educators

The perceptions of the principals demonstrated that clinical facilities that facilitate learning for learner nurses pose various challenges relating to the new programmes’ implementation. These challenges include limited sites for midwifery clinical learning and a shortage of clinical mentors or preceptors who provide clinical support to the learner nurses in different clinical facilities. The following extracts support the interpretation:

‘The clinical site for the midwifery challenge is not having enough deliveries to cater to both college and university students …’ (Participant 1, 46 years, female)

‘Challenges include a shortage of nursing personnel, requiring educators to accompany students, struggling with script marking, and scattered students in hospitals.’ (Participant 5, 57 years, female)

‘We don’t have transport to clinical practice.’ (Participant 6, 64 years, male)

### Theme 2: Infrastructural and material constraints

Participants mentioned material and infrastructural resource issues affecting the realisation of the Fourth Industrial Revolution (4IR) and implementation of the new qualifications in NEIs. Other issues included limited libraries, computer laboratories, kitchens and student accommodation.

#### Sub-theme 2.1: Infrastructure-related challenges

The principals proffered that the infrastructure-related challenges impacting adversely on implementing the new academic nursing programme included limited library access or space and small classrooms compared to the quota of admitted learner nurses. To support these findings, refer to the following extracts from principal participants:

‘We have one library which cannot accommodate a large number of students … Classrooms are small for a large number of students.’ (Participant 1, 46 years, female)

‘Our institution faces significant infrastructural and material resource gaps, including a small library with limited capacity for 10–12 students and no separate study centre.’ (Participant 8, 61 years, female)

‘The campus, with small classroom sizes, has undergone renovations since 2014, but the process is still incomplete, posing challenges for students’ accommodation.’ (Participant 6, 64 years, male)

#### Sub-theme 2.2: Material-related challenges

The materials that support teaching and learning were regarded as a challenge by some principals. Some mentioned purchasing books, the need for gadgets and low-fidelity teaching models as challenges that can impede the implementation of the new programme. We extracted the following quotes to support these findings:

‘We have only old books dated back to 2010. We did not purchase any e-books the only book that the students could access through their cellphones it’s a psyche book.’ (Participant 3, 57 years, female)

‘We also need a model gadget, computers, and e-learning programmes, and to convert another classroom to establish a computer laboratory for our students.’ (Participant 6, 64 years, male)

‘We have only low-fidelity teaching models and projectors. We sent our outstanding needs list to the Department of Health, and they agreed to address it, so we are hopeful that we will have at least the basic resources to begin the programme.’ (Participant 8, 61 years, female)

#### Sub-theme 2.3 Technology-related problems to realise the Fourth Industrial Revolution

The participants referred to technology-related issues such as shortages of learning and teaching devices, Internet and wireless fidelity (WiFi) access, as well as a lack of a student information system for supporting e-learning. The following excerpts attest to the participants’ perspectives in this regard:

‘We do not have a student management system, and I believe there are none at all of the Public Nursing Colleges throughout South Africa. Our computer centre can only take 20 computers.’ (Participant 8, 61 years, female)

‘No e-books established as yet. The IT challenge includes WiFi due to financial constraints.’ (Participant 4, 53 years, female)

‘Outsourced service provider focuses on digitalizing learner management info system, aiming for an online recruitment process, computerized selection, and complete learner registration to streamline selection and candidate selection.’ (Participant 13, 58 years, male)

### Theme 3: Clinical services and logistical problems that could affect the implementation

The participants made reference to logistical and service problems that could negatively affect the implementation of the new programmes in the NEIs. These challenges entailed the shortage of clinical area mentors, high-fidelity models and deliveries for midwives.

#### Sub-theme 3.1: Management of Information System concerning the implementation of new qualifications

The participants mentioned problems regarding the management of information systems (MIS) in conjunction with the new qualifications. While noting the importance of having a student information system within the NEIs, there seems to be a serious challenge since most of the colleges reported a lack of proper recording systems of students’ information. The following extracts from the participants support these findings:

‘The student information system is the key issue. If it is not available, we may not be accredited.’ (Participant 1, 46 years, female)

‘We have serious challenges because our student affairs personnel consists of only, one student clerk. We do manual filing. We have one archive that was flooded the other day. The CNO has been in communication with the relevant department to try and set up this system nationally.’ (Participant 8, 61 years, female)

‘For record-keeping, there is the registry office which is unoccupied at the moment. The filing sections are utilized by HR. It is always locked, and their records are labelled.’ (Participant 10, 64 years, female)

#### Sub-theme 3.2: Institutional challenges at the initial and working stages of curriculation of new programmes

The participants mentioned challenges encountered in the initial and actual stages of recurriculating the new programmes. Some of the participants reported a need for a workshop or training on the development of the new programme and were also given a sample of a national programme from which to refer when designing it for their institution. The following extracts support these findings:

‘I feel that there is still a need for more workshops, especially with clarification of new concepts, and the approach, and this was spotted as there was a lot of discussion and debate. I think the educators still need some workshops to thoroughly understand the changes involved.’ (Participant 10, 64 years, female)

‘We were under the impression that we would be given the national curriculum which did not happen, and we were informed that we should start curriculating as provinces. This posed a challenge as we are operating as four independent colleges.’ (Participant 13, 58 years, male)

‘The process was marked by trial and error, resulting in lost resources, money, and time. Challenges included educator attrition without replacement and the ongoing process of preparing for the postgraduate diploma.’ (Participant 5, 57 years, female)

### Theme 4: Mixed perception of support provided to public nursing colleges in preparation for the implementation of the new qualifications

Participants showed mixed perceptions of support from collegial, management, provincial DoH, CHE and SNC during the implementation of the new programme. These perceptions ranged from adequate, limited or no support for study guides, curricula, staff categories and staff development at various levels.

#### Sub-theme 4.1: Support from bodies responsible for accreditation (Department of Health, South African Nursing Council, Council on Higher Education, Nursing/Midwifery Education Partnership Initiative (NEPI), and Chief Nursing Officer)

The participants mentioned different support levels by the SANC, CHE, NEPI, CNO and provincial DoH. Some participants cited adequate support, while others mentioned limited support, with the rest indicating that they were not provided with support. There was support for developing staff and study guides, increasing different staff categories, as well as curriculation. The participants mentioned the following in support:

‘The team attended national curriculum meetings to support lecturers’ training, with coordinators coordinating workshops, meetings, updates, and mentoring progress across all provinces.’ (Participant 1, 46 years, female)

‘The CHE provided support to colleges in uploading curriculum, holding regular principal meetings, compiling a state of readiness report, and conducting a workshop in 2016.’ (Participant 4, 53 years, female)

‘We haven’t had any contact with the accrediting bodies so I can’t say we have support.’ (Participant 8, 61 years, female)

## Discussion of the findings

The paper aimed at exploring the challenges attendant to implementation of new qualifications in nursing at selected PNCs in four provinces (North West, Gauteng, Limpopo, and Free State). Makhanya (2022) noted that lecturers at all campuses comply with the requirements of the CHE regarding the prioritisation of programmes. Nursing colleges face challenges because of an ageing population of nurse educators, leading to a loss of competence and institutional memory. Mochaki (2018) emphasised that professional development is critical for the continual learning and growth of nurse educators. There is a need to design programmes aimed at developing capacity and for replacing positions, but implementation is deficient. Nurse educators need collaboration and professional development regrading programme design and implementation.

Abdullahi et al. (2019) highlighted that the nursing profession’s development in Nigeria was hampered mainly by poor staffing or shortage of qualified faculty members. Similarly, nurses in Lesotho experienced challenges in implementing a new technology-driven curriculum that also encompassed simulations (Botma 2014). Consequently, the range of challenges has resulted in NEIs often recruiting or luring educators from each other’s campuses or re-employing those who retired on a contract basis instead of promoting educators who are newly qualified educators (Blaauw et al. 2014). This practice is justified in part by the lack of replacements for retiring educators because of the health sector’s financial limitations and government’s reluctance to create new positions in nursing.

Netshiswinzhe Mcur and Mulaudzi (2015) observed that the OSD, a competitive process, could exacerbate the shortage of nurses in some institutions. According to George, Atujuna and Gow (2013), well-known causes of the shortage of nurse educators include nurse migration because of OSD and rural allowances, nurse retirees who are not replaced, and a newly qualified cohort of educators to teach new programmes.

Mogano (2016) recognised the need to transition from old to new programmes to address critical nursing shortages. Transferring NE to the DHET was still pending because of amendments to the *Nursing Act* that were awaited. Furthermore, for new programmes to succeed, newly qualified professionals need support through mentorship or preceptorship programmes. The current study’s findings align with Barret and Treves (2019), who reported that infrastructure challenges often hinder the quality of programme implementation and learning. Mathase (2021) also noted that inadequate infrastructure in educational institutions negatively affects both teachers and learners.

Abdullahi et al. (2019) pointed out that poor funding has led to a lack of materials, affecting nursing programme development in Nigeria. Direko and Davhana-Maselesele (2017) suggested that the nursing profession’s resource challenges could be mitigated by fostering participation, interdependence, and resource-sharing through collaboration between colleges, hospitals, and universities. The role of technology in NE has been widely discussed, with Ralph et al. (2014) emphasising its impact in Australian healthcare settings, while Kovaleski et al. (2021) underscored the importance of skills and knowledge related to e-health technologies. These authors also highlighted that technologies such as mobile devices, telehealth and telemedicine have transformed healthcare delivery and are pivotal to nursing national strategies. In Lesotho, nursing curricula incorporated information and communication technology (ICT) training and facilities, supported by NEPI’s establishment of simulation and computer laboratories (Botma 2014).

The use of Learning Management Systems (LMS) is gathering traction in NE although their implementation with blended learning remains inconsistent (Sáiz-Manzanares, Escolar-Lamazares & González 2020). The primary author suggests that LMS could be supportive of implementing new programmes in nursing. Similarly, Posey and Pintz (2017) noted that colleges have the potential to use LMS for scholarly development among students and educators. However, the CHE (2000) reported that information systems in South African higher education remain insufficient. Proper record management is essential to help institutions manage information, preserve corporate memory and ensure accountability and good governance. Blaauw et al. (2014) and Zwane and Mtshali (2019) found that unclear and inconsistent policy direction regarding the transition to higher education, poor stakeholder communication and inadequate clinical learning support have resulted in declining learning standards. These issues were compounded by unclear transitional arrangements and a lack of collaborative planning.

In Nigeria, nursing development was supported by the National Board of Technical Education, National Universities Commission and the National Commission of Colleges for Education (Abdullahi et al. 2019). However, the profession was still affected by inadequate government support. In South Africa, the SANC and CHE played crucial roles in integrating provincial nursing colleges into higher education (Zwane & Mtshali 2019). They further noted that partnerships between government, healthcare organisations and academic institutions were key to resolving curriculum challenges and training in both clinical and academic settings. Support for PNCs in this transition is critical for the successful implementation of new qualifications.

### Limitations

The study adopted only a qualitative research approach in response to the research question. As such, it is not feasible to generalise the findings that attempted the same challenge but with a different research approach. The study was only conducted on selected NEIs, and generalising the findings to other nursing colleges might not make it easy to implement new programmes.

### Recommendations

Nurse educators’ ageing population necessitates capacity-building programmes, including succession planning, professional development and mentorship, to preserve knowledge and institutional memory. Regulation 174, which governs nursing qualifications and educator capacity, requires programmes to fully accommodate it to maintain compliance and ensure consistency in training across nursing institutions. For the purpose of fostering collaboration between NEIs, healthcare providers and universities, there should be sharing of resources, expertise and best practices; alleviation of financial constraints and improvement of resource availability.

Governments should focus on recruiting and retaining new nurse educators by offering competitive salaries and career development opportunities, rather than relying on retirees or staff from other institutions. Nursing curricula should incorporate modern technologies such as simulation laboratories, telehealth and e-learning platforms, enhancing teaching and learning experiences and making NE more adaptable to global healthcare needs. The LMS and blended learning models are crucial for enhancing teaching efficiency and professional development in nursing, offering flexible learning opportunities and supporting the rollout of new qualifications.

Collaboration between the SANC, CHE and government agencies is crucial for strengthening government support for NE reform, ensuring smooth transitions and addressing staffing shortages. New nursing graduates require preceptorship or mentorship programmes for structured support and preparation for modern healthcare challenges.

### Measures to Ensure Trustworthiness

The primary author ensured the study’s credibility by building trust and rapport with principals through prolonged engagement during interviews and triangulating data from different study sites (Connelly 2016). Non-verbal cues were also observed and noted. Data authentication involved the primary and secondary authors, as well as an independent coder supervisor (Connelly 2016). Interview recordings and transcripts were securely stored, accessible only to the authors and the coder. To maintain objectivity, the primary author refrained from expressing personal views and held a consensus meeting with the independent coder on the study’s themes. The study’s background, design, methodology, data collection and analysis were clearly explained.

## Conclusion

The current study findings create awareness among the SANC, CHE and DoH about the progress and the state of the new nursing programme implementation. The perspective illustrates various challenges that can be categorised into human, institutional and structural factors. These challenges suggest a need to strengthen the academic support and collaboration between internal and external stakeholders of NEIs for effectively implementing the new programmes in nursing. We recommend a model development for supporting the implementation of the new programmes in nursing across South Africa.
